# Abnormal insulin-like growth factor 1 signaling in human osteoarthritic subchondral bone osteoblasts

**DOI:** 10.1186/ar2087

**Published:** 2006-11-27

**Authors:** Frédéric Massicotte, Isabelle Aubry, Johanne Martel-Pelletier, Jean-Pierre Pelletier, Julio Fernandes, Daniel Lajeunesse

**Affiliations:** 1 Unité de recherche en arthrose, Centre de recherche du centre hospitalier de l'Université de Montréal, Hôpital Notre-Dame, Montréal, Québec, Canada; 2 Centre de recherche, Hôpital Sacré-Cœur, Montréal, Québec, Canada

## Abstract

Insulin-like growth factor (IGF)-1 is a key factor in bone homeostasis and could be involved in bone tissue sclerosis as observed in osteoarthritis (OA). Here, we compare the key signaling pathways triggered in response to IGF-1 stimulation between normal and OA osteoblasts (Obs). Primary Obs were prepared from the subchondral bone of tibial plateaus of OA patients undergoing knee replacement or from normal individuals at autopsy. Phenotypic characterization of Obs was evaluated with alkaline phosphatase and osteocalcin release. The effect of IGF-1 on cell proliferation, alkaline phosphatase and collagen synthesis was evaluated in the presence or not of 50 ng/ml IGF-1, whereas signaling was studied with proteins separated by SDS-PAGE before western blot analysis. We also used immunoprecipitation followed by western blot analysis to detect interactions between key IGF-1 signaling elements. IGF-1 receptor (IGF-1R), Shc, Grb2, insulin receptor substrate (IRS)-1, and p42/44 mitogen-activated protein kinase (MAPK) levels were similar in normal and OA Obs in the presence or absence of IGF-1. After IGF-1 stimulation, the phosphorylation of IGF-1R in normal and OA Obs was similar; however, the phosphorylation of IRS-1 was reduced in OA Ob. In addition, the PI3K pathway was activated similarly in normal and OA Obs while that for p42/44 MAPK was higher in OA Obs compared to normal. p42/44 MAPK can be triggered via an IRS-1/Syp or Grb2/Shc interaction. Interestingly, Syp was poorly phosphorylated under basal conditions in normal Obs and was rapidly phosphorylated upon IGF-1 stimulation, yet Syp showed a poor interaction with IRS-1. In contrast, Syp was highly phosphorylated in OA Obs and its interaction with IRS-1 was very strong initially, yet rapidly dropped with IGF-1 treatments. The interaction of Grb2 with IRS-1 progressively increased in response to IGF-1 in OA Obs whereas this was absent in normal Ob. IGF-1 stimulation altered alkaline phosphatase in Ob, an effect reduced in the presence of PD98059, an inhibitor of p42/44 MAPK signaling, whereas neither IGF-1 nor PD98059 had any significant effect on collagen synthesis. In contrast, cell proliferation was higher in OA Obs compared to normal under basal conditions, and IGF-1 stimulated more cell proliferation in OA Obs than in normal Ob, an effect totally dependent on p42/44 MAPK activiy. The altered response of OA Obs to IGF-1 may be due to abnormal IGF-1 signaling in these cells. This is mostly linked with abnormal IRS-1/Syp and IRS-1/Grb2 interaction in these cells.

## Introduction

Osteoarthritis (OA) represents a major cause of disability, particularly among the aging population; indeed, it is the most common form of joint disease. OA is a multifactorial disease characterized by loss of articular cartilage and subchondral plate thickening [1]. As the loss of articular cartilage is believed to be the initial event responsible for joint destruction, numerous investigations have focused their efforts on understanding cartilage homeostasis. Therefore, biochemical analysis of the underlying bone tissue has received little attention, despite several reports of abnormal subchondral bone metabolism in OA.

Radin and coworkers were the first to study subchondral bone changes in patients with early degenerative joint disease, and to propose the participation of subchondral bone in the initiation and progression of cartilage damage [2,3]. They proposed that the thickening of the subchondral bone plate, resulting from repeated healing of microfractures, could be a key initiation factor in cartilage degradation. Other groups also reported abnormal cancellous bone collagen metabolism in OA, demonstrating that type I collagen, the most abundant bone matrix protein, is abnormal in OA [4,5]. Recently, we demonstrated that osteoblast (Ob) cells from human subchondral OA bone demonstrate an altered phenotype *in vitro*. Our results showed increased alkaline phosphatase activity, release of osteocalcin, an increased activity of urokinase plasminogen activator (uPA) with no changes in plasminogen activator inhibitor-1 (PAI-1) abundance, and increases in insulin-like growth factor (IGF)-1 release compared to normal Obs [6-8]. As IGF-1 production is increased in OA Obs compared to normal Obs, it is a likely candidate to promote bone remodeling and sclerosis in OA. Interestingly, our laboratory also demonstrated the presence of abnormal uPA regulation by IGF-1 in human OA Obs [6]. These results suggest that IGF-1 signaling could be altered in these cells [6]. The increased remodeling in OA bone could possibly account for the observation of hypomineralization of the subchondral bone tissues in established OA [9-11]. Not only the bone matrix is altered in OA but recent studies have demonstrated that a putative factor(s) produced by OA subchondral bone cells can influence cartilage metabolism [12]. This could possibly explain why increased subchondral bone activity can predict cartilage loss [13-15].

After binding of IGF-1 to its specific surface receptor, the IGF-1 receptor (IGF-1R) kinase undergoes tyrosine phosphorylation of its α-subunit and kinase activation. This involves the phosphorylation of tyrosine residues of substrate adaptor proteins, principally the insulin receptor substrate (IRS)-1. Other targets have also been identified, such as Shc, IRS-2, IRS-3 and IRS-4 and GAB1 [16,17]. These proteins contain insulin/IGF-1R-specific tyrosine phosphorylation sites responsible for their association with various SH2 domain-containing downstream effector molecules. In the case of IRS-1, these include binding sites for phosphatidylinositol 3-kinase (PI3K), protein tyrosine-specific phosphatase Syp, 14.3.3 proteins, and the small adaptor protein Grb2, which is responsible for the activation of Ras and the MAPK pathway [18,19].

Thus, as the response to IGF-1 in human OA Obs is abnormal, we investigated IGF-1 signaling in OA Obs. Data revelead an abnormal interaction of phospho-Syp with IRS-1, possibly leading to decreased IRS-1 activity. Moreover, the interaction of Grb2 with IRS-1 was abnormal in OA Obs, possibly leading to altered downstream signaling. These data suggest that an abnormal response to IGF-1 is linked to abnormal intracellular signaling, affecting multiple pathways in OA osteoblasts.

## Materials and methods

### Patients and clinical parameters

Subchondral bone was obtained from OA patients who had undergone total knee replacement surgery. Specifically, medial tibial plateaus were dissected away from the remaining cartilage and trabecular bone under sterile conditions as previously described [6-8,20]. Only the middle portion of the tibial plateaus was used to separate the subchondral bone plate specimens. A total of 40 patients (aged 70.8 ± 7.9 years (mean ± standard deviation (SD)); 18 males, 22 females) classified as having OA according to recognized American College of Rheumatology clinical criteria were included in this study [21]. None of the patients had received medication that would interfere with bone metabolism, including corticosteroids, for six months before surgery. A total of 16 bone specimens of medial tibial plateaus from normal individuals (aged 61.5 ± 15.6 years (mean ± SD); 9 males, 7 females) were collected at autopsy within 12 h of death. These were used following the establishment that the donors had not been on any medication that could interfere with bone metabolism or had any bone metabolic disease. Individuals showing cartilage deterioration and/or subchondral bone plate sclerosis were not included in the normal group. All human material was acquired following a signed agreement by patients undergoing knee surgery or their relatives for the specimens collected at autopsy following the Centre Hospitalier de l'Université de Montréal (CHUM) ethical committee guidelines.

### Preparation of primary bone cell culture

The subchondral bone plate was dissected away from the remaining cartilage and trabecular bone under sterile conditions. Isolation of subchondral bone plate and the cell cultures were prepared as previously described by a collagenase digestion procedure [6,7,22,23]. At confluence, cells were passaged once at 25,000 cells/cm^2 ^in 6 well plates and grown for 5 days in Ham F12/DMEM (Sigma, St-Louis, MO, USA) containing 10% FBS (Wisent Inc., St Bruno, Quebec, Canada) before specific assays. Under these culture conditions, Obs expressed bone-specific type I collagen without any contaminations with cartilage-specific type II collagen [23]. Conditioning was performed for an additional 24 h in serum free Ham F12/DMEM media. Confluent cells were incubated or not with human IGF-1 (50 ng/ml; Peninsula, Belmont, CA, USA) for different times as specified per individual experiments. In some experiments, PD98059 (Sigma-Aldrich), an inhibitor of the MAP-kinase/Erk kinase pathway, was used at a final concentration of 10 μM in the presence or absence of IGF-1, and controls were treated with the vehicle. Supernatants were collected at the end of the incubation and kept at -80°C prior to assays. Cells were lysed with RIPA buffer (50 mM Tris-HCl pH 7.4, 1% NP-40, 0.5% Na-deoxicholate, 0.1% SDS, 150 mM NaCl, with the inhibitors 10 μg/ml aprotinin, 10 μg/ml leupeptin, 10 μg/ml pepstatin, 10 μg/ml O-phenatroline, 1 mM Na-orthovanadate, 1 mM dithiothreitol), and kept at -80°C prior to assays. Protein determination was performed by the bisinchoninic acid method [24].

### Western immunoblotting and immunoprecipitation

The cell lysates were loaded on polyacrylamide gels and separated by SDS-PAGE under reducing conditions [25]. Loading was adjusted according to the cellular protein concentration of each specimen and western blotting of actin was performed to assess similar loading between samples. The proteins were electrophoretically transfered onto PVDF western blotting membranes (Boehringer Mannheim, Penzberg, Germany). Immunoblotting was performed as described in the ECL Plus Western blotting detection system's manual (Amersham Pharmacia Biotech, Baie d'Urfe, Québec, Canada) using a variety of primary and secondary antibodies. Primary antibodies included: IGF-1R α-subunit (ab-5, 1:10,000 dilution) from NeoMarkers (Fremont, CA, USA); phosphorylated IGF-1R (pY1158, 1:10,000 dilution) from Biosource International (Camarillo, CA, USA); IRS-1, IRS-2, Grb2 and PI3K (1:2,000 dilutions) from Upstate Biotechnology (Lake Placid, NY, USA); phospho IRS-1 (pS307, 1:1,000 dilution) from Upstate Biotechnology; phosphotyrosine Ab-1 (Clone PY-20, 1:15,000 dilution) from NeoMarkers; Shc, and Gab1 (1:5,000 dilutions) from Santa Cruz Biotechnology (Santa Cruz, CA, USA); phosphorylated Shc (Tyr317, 1:10,000 dilution), protein kinase B (PKB), phosphorylated PKB (Ser473, 1:1,000 dilution), p42/44 and phosphorylated p42/44 (Thr202/Tyr204, 1:5,000 dilutions) from Cell Signaling Technology (Beverly, MA, USA); Syp from BD Transduction Laboratories (Mississauga, ON, Canada); and actin (1:10,000 dilution) from Sigma-Aldrich. Secondary antibodies included: goat anti-mouse IgG (1:100,000 dilution) from Pierce (Rockfort, IL, USA); rabbit anti-sheep IgG (1:20,000 dilution) and goat anti-rabbit IgG (1:10,000 dilution) from Upstate Biotechnology. The immunoprecipitation was performed with 175 μg of the solubilized cell extracts incubated with 4 μg of IRS-1 or 0.5 μg of IGF-1R α-subunit (Ab-4) antibody, overnight at 4°C. The resulting immunoprecipitates were subjected to SDS-PAGE as described above. Densitometry analysis of western blot films was performed on a Macintosh Mac OS 9.1 computer using the public domain NIH Image program developed at the US National Institutes of Health with the Scion Image 1.63 program [26].

### Real-time RT-PCR quantification

Real-time quantification of Bax-α, Bcl2 and glyceraldehyde-3-phosphate dehydrogenase (GAPDH) mRNA from normal and OA Obs treated for 6 h with 100 ng/ml IGF-1 was performed in the GeneAmp 5700 Sequence Detection System (Applied Biosystems, Foster City, CA, USA) with the 2× Quantitect SYBR Green PCR Master Mix (Qiagen, Mississauga, ON, Canada) used according to the manufacturer's specifications. In brief, 100 ng of the cDNA obtained from the RT-PCR reactions were amplified in a total volume of 50 μl consisting of 1× Master Mix, 0.5 Unit uracil-N-glycosylase (UNG; Epicentre Technologies, Madison, WI, USA) and the gene-specific primers added at a final concentration of 200 nM. The specific primers were: for Bax-α, 5'-GGA TGC GTC CAC CAA GAA G-3' (sense) and 5'-CAC CAG TTT GCT GGC AAA G-3' (antisense); and for Bcl2, 5'-GGC ATC TTC TCC TCC CAG C-3' (sense) and 5'-GAA GGG CGT CAG GTG CA-3' (antisense); these generated fragments of 208 base-pairs (bp) and 202 bp, respectively. To ensure equivalent loading, GAPDH was amplified using 20 pmol of each of the primers 5'-CAG AAC ATC ATC CCT GCC TCT-3' (sense) and 5'-GCT TGA CAA AGT GGT CGT TGAG-3' (antisense) to generate a predicted amplified sequence of 318 bp [27]. The tubes were first incubated for 2 minutes at 50°C (UNG reaction), then at 95°C for 15 minutes (UNG inactivation and polymerase activation) followed by 40 cycles consisting each of denaturation (94°C for 15 s), annealing (60°C for 30 s), extension (72°C for 30 s) and data acquisition (77°C for 15 s) steps. The data were collected and processed with the GeneAmp 5700 SDS software (Applied Biosystems, Foster City, CA, USA) and given as threshold cycle (Ct), corresponding to the PCR cycle at which an increase in reporter fluorescence above baseline signal can first be detected. Plasmid DNAs containing the target gene sequences were used to generate the standard curves. When comparing normal and OA expression levels, the threshold cycle (Ct) was converted to number of molecules and the values for each sample calculated as the ratio of the number of molecules of the target gene/number of molecules of GAPDH.

### Phenotypic characterization of human subchondral osteoblast cell cultures

Phenotypic features of Obs cultures were determined by evaluating 1,25(OH)_2_D_3_-dependent (50 nM) alkaline phosphatase activity and osteocalcin release, and by the production of collagen type 1. Alkaline phosphatase activity was determined on cell aliquots by substrate hydrolysis using p-nitrophenylphosphate, and osteocalcin release was determined in cell supernatants using an enzyme immunoassay as previously described [7,20]. Collagen synthesis was determined as the *de novo *release of the carboxy-terminal peptide fragment (CICP) of collagen type 1, reflecting true collagen synthesis. This fragment was determined using a selective enzyme-linked immunosorbent assay (ELISA; Quidel Corporation, Cederlane, Hornby, ON, Canada) in conditioned media from confluent Obs incubated in Ham F12/DMEM media containing 0.5% BSA. Cellular proliferation was assessed using the bromodeoxyuridine (BrdU) cell proliferation assay as described in the system's manual from Calbiochem (San Diego, CA, USA). Cells were plated at 10,000 cells/cm^2 ^in 96-well plates in Ham F12/DMEM media containing 10% FBS. After overnight attachment, cells were serum-starved in Ham F12/DMEM media containing 0.5% BSA for 24 h prior to stimulation with or without 50 ng/ml IGF-1 in the presence or absence of 10 μM PD98059 in the same media.

### Statistical analysis

All quantitative data are expressed as mean ± SEM. The data were analyzed by Student's *t*-test, and p values < 0.05 were considered statistically significant.

## Results

### Phenotypic characteristics of normal and OA osteoblasts and response to IGF-1

The phenotypic characteristics of normal and OA Obs were first determined by the production of alkaline phosphatase and release of osteocalcin. Values for normal Obs were 575.6 ± 89.4 nmol/mg protein/30 minutes and 141.6 ± 15.5 ng/mg protein for alkaline phosphatase and osteocalcin, respectively. These values were increased in OA Obs and reached 1259.2 ± 158.0 nmol/mg protein/30 minutes (p < 0.01 versus normal) and 261.2 ± 23.5 nmol/mg protein (p < 0.005 versus normal) for alkaline phosphatase and osteocalcin, respectively, as previously described [6-8]. OA osteoblasts also synthesized more collagen type I than normal Obs (3254 ± 272 versus 4246 ± 189 for normal and OA, respectively, p < 0.01) whereas they failed to produce collagen type II as previously shown [20].

**Figure 1:**
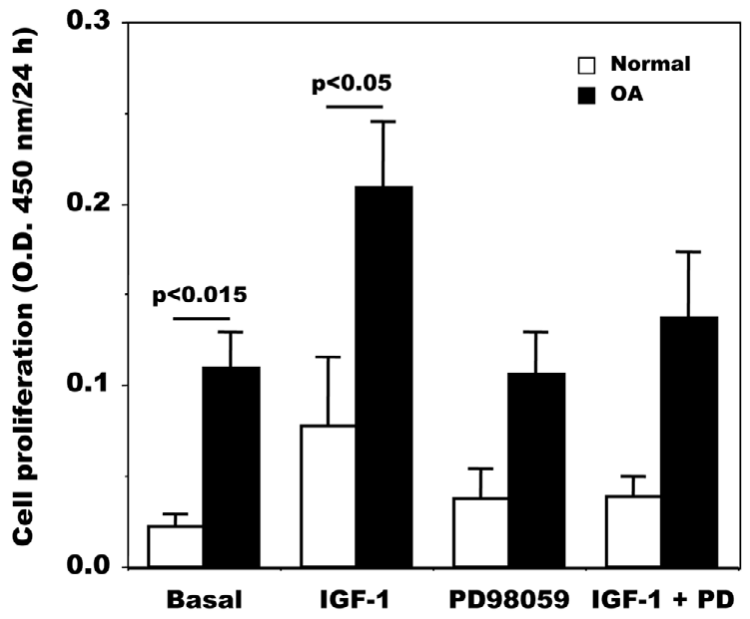
Cell proliferation of normal and osteoarthitis (OA) osteoblasts in response to insulin-like growth factor (IGF)-1 stimulation. Cells were plated at 10,000 cells/cm^2 ^in 96-well plates and incubated overnight in Ham F12/DMEM media containing 10% FBS. Cells were then serum-starved for 24 hours in the same media containing 0.5% BSA then treated with or without 50 ng/ml IGF-1 in the presence or not of 10 μM PD98059 (PD) for 24 hours followed by incubation with bromodeoxyuridine (BrdU) for their last 24 hours of incubation in the same media. BrdU incorporation was then evaluated following the manufacturer's instructions. Results are expressed as mean OD units ± SEM for three normal and four OA osteoblast preparations.

**Figure 2:**
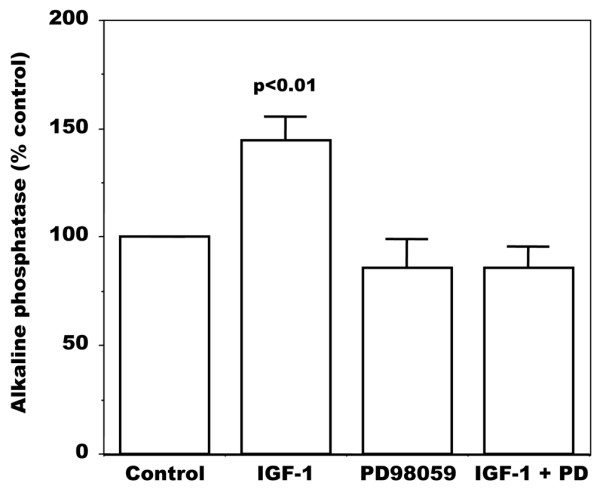
Effect of insulin-like growth factor (IGF)-1 on alkaline phosphatase activity in osteoarthitis (OA) osteoblasts. Cells were grown to confluence and incubated overnight in serum free medium. Cells were then exposed to 50 nM 1,25(OH)_2_D_3 _in Ham F12/DMEM media containing 2% charcoal-treated FBS and in the presence or not of 50 ng/ml IGF-1 with or without 10 μM PD98059 (PD). Results are expressed as the mean ± SEM of control values without IGF-1 for 6 OA osteoblast preparations.

Cell proliferation was higher in OA Obs compared to normal Obs (p < 0.015), and IGF-1 promoted cell proliferation in both normal and OA Obs while the effect was more important in OA Obs than normal Obs (Figure 1). This effect of IGF-1 was blocked by PD98059, an inhibitor of the MAPK/Erk-kinase pathway, and cell proliferation rate returned to basal values. IGF-1 stimulation also increased alkaline phosphatase activity in both normal and OA Obs. In normal Obs, IGF-1 raised alkaline phosphatase activity from 504.9 ± 83 nmol/mgprotein/30 minutes to 625.4 ± 36.1 (23.9% increase) whereas this activity in OA Obs increased from 712.9 ± 154.9 to 1099.4 ± 346.6 nmol/mg protein/30 minutes (44.8% increase). This effect was blocked by PD98059 in OA Obs (Figure 2). However, IGF-1 failed to stimulate collagen type I synthesis in both normal and OA Obs, and PD98059 was without effect on this parameter (not illustrated). We also evaluated the expression of Bax-α and Bcl2 to evaluate the apoptotic potential of these cells [28-30]. Using quantitative RT-PCR we were able to demonstrate a reduction in the Bax-α to Bcl2 ratio in OA Obs compared to normal. Indeed, the ratio of Bax-α to Bcl2 was 22.6 ± 3.4 versus 12.2 ± 2.4 relative units in normal (*n *= 3) and OA Obs (*n *= 3), respectively (p < 0.05).

### IGF-1R levels and activation are similar in normal and osteoarthitis osteoblasts

**Figure 3:**
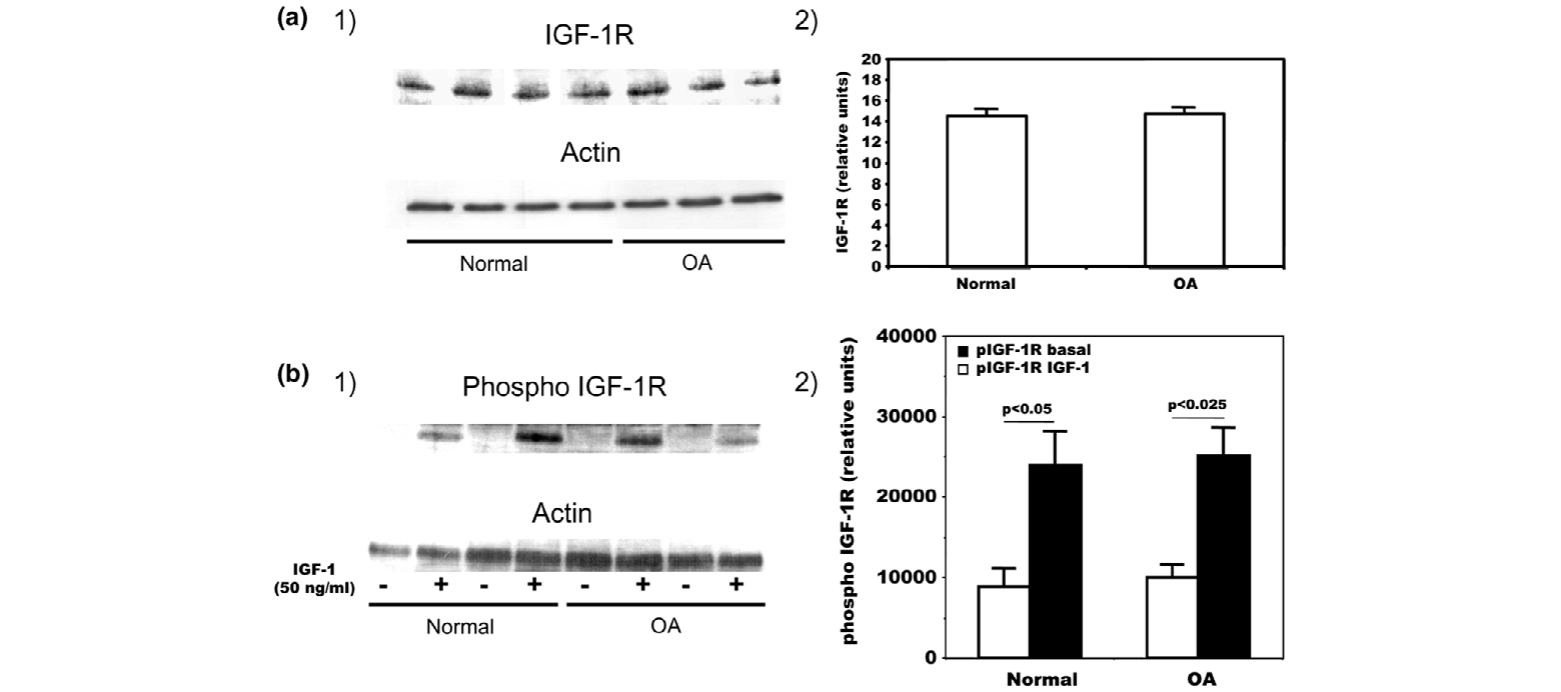
Insulin-like growth factor (IGF)-1 receptor (IGF-1R) levels and activation in normal and osteoarthitis (OA) osteoblasts. Cells were grown to confluence and incubated overnight in serum free medium. **(a) **After cell lysis in RIPA buffer and immunoprecipitation with an anti-IGF-1R α-subunit antibody (ab 4), receptor levels were analyzed by western immunoblot with an anti-IGF-1R α-subunit antibody (ab 5). **(b) **After cell lysis in RIPA buffer, tyrosine phosphorylation of the receptor exposed to 50 ng/ml IGF-1 for 5 minutes was analyzed with anti-IGF-1R phosphotyrosine ab (PY-1158). Determination of actin was used as the control for loading. Representative data are shown in panel 1 (a,b). Results expressed as arbitrary scanning units are presented as the mean ± SEM (panel 2 (a,b)).

IGF-1R levels in normal and OA Obs were evaluated by western immunoblot analysis. As presented in Figure 3a, no significant difference could be observed between normal (*n *= 5) and OA (*n *= 5) Obs IGF-1R levels under basal conditions. The phosphorylation of IGF-1R following IGF-1 stimulation was very rapid and similar in normal (*n *= 3) and OA (*n *= 3) Obs and increased 2.86 ± 0.36 and 2.52 ± 0.06-fold, respectively, above basal levels after 30 s (Figure 3b), and phosphorylated IGF-1R levels remained similar until 5 minutes of stimulation with IGF-1 (data not shown).

### IRS-1 is underphosphorylated in osteoarthitis osteoblasts

**Figure 4:**
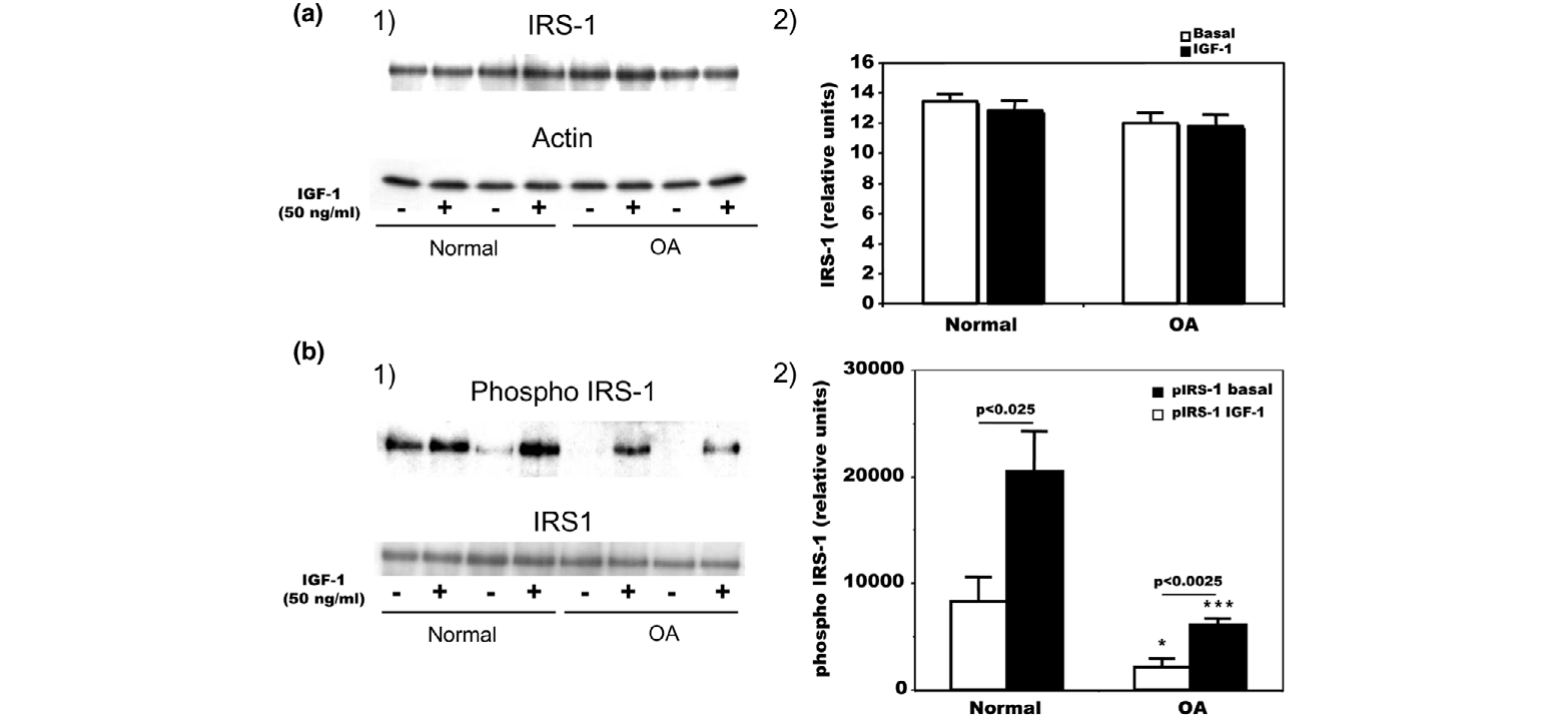
Insulin receptor substrate (IRS)-1 levels and activation in normal and osteoarthitis (OA) osteoblasts. Cells were grown to confluence and incubated overnight in serum free medium. Cells were treated with 50 ng/ml insulin-like growth factor (IGF)-1 for 5 minutes. **(a) **After cell lysis in RIPA buffer and immunoprecipitation with anti-IRS-1 antibodies, IRS-1 levels were analyzed by immunoblotting with the same anti-IRS-1 antibody. **(b) **After cell lysis in RIPA buffer and immunoprecipitation with an anti-IRS-1 antibody, phosphorylated IRS-1 levels were analyzed by immunoblotting with an anti-phosphotyrosine antibody (PY-20). Determination of actin was used as the control for loading. Representative data are shown in panel 1 (a,b). Results expressed as arbitrary scanning units are presented as the mean ± SEM (panel 2 (a,b)).

Figure 4a shows that IRS-1 levels in normal (*n *= 4) and OA (*n *= 4) Obs were similar. However, the tyrosine phosphorylation of IRS-1 was significantly altered in OA Obs compared to normal. Under basal conditions, the tyrosine phosphorylation of IRS-1 in OA Obs was 4.65 ± 1.24-fold less phosphorylated than normal (p < 0.05), and after a 5 minute stimulation with IGF-1, this level was still about 3.52 ± 0.35-fold less phosphorylated (p < 0.005; Figure 4b). Serine phosphorylation of IRS-1 in OA Obs was similar to normal (data not shown). As we were unable to detect clear levels of IRS-2 in our Obs, we excluded the possibility that increasing IRS-2 activation could lead to downregulation of IRS-1 (data not shown). Moreover, 14.3.3 protein, which is known to bind IRS-1 and modulate its activation, was not significantly different between normal and OA Obs (data not shown).

### p42/44 phosphorylation are upregulated while PI3K/PKB pathway is unaltered in OA Obs

**Figure 5:**
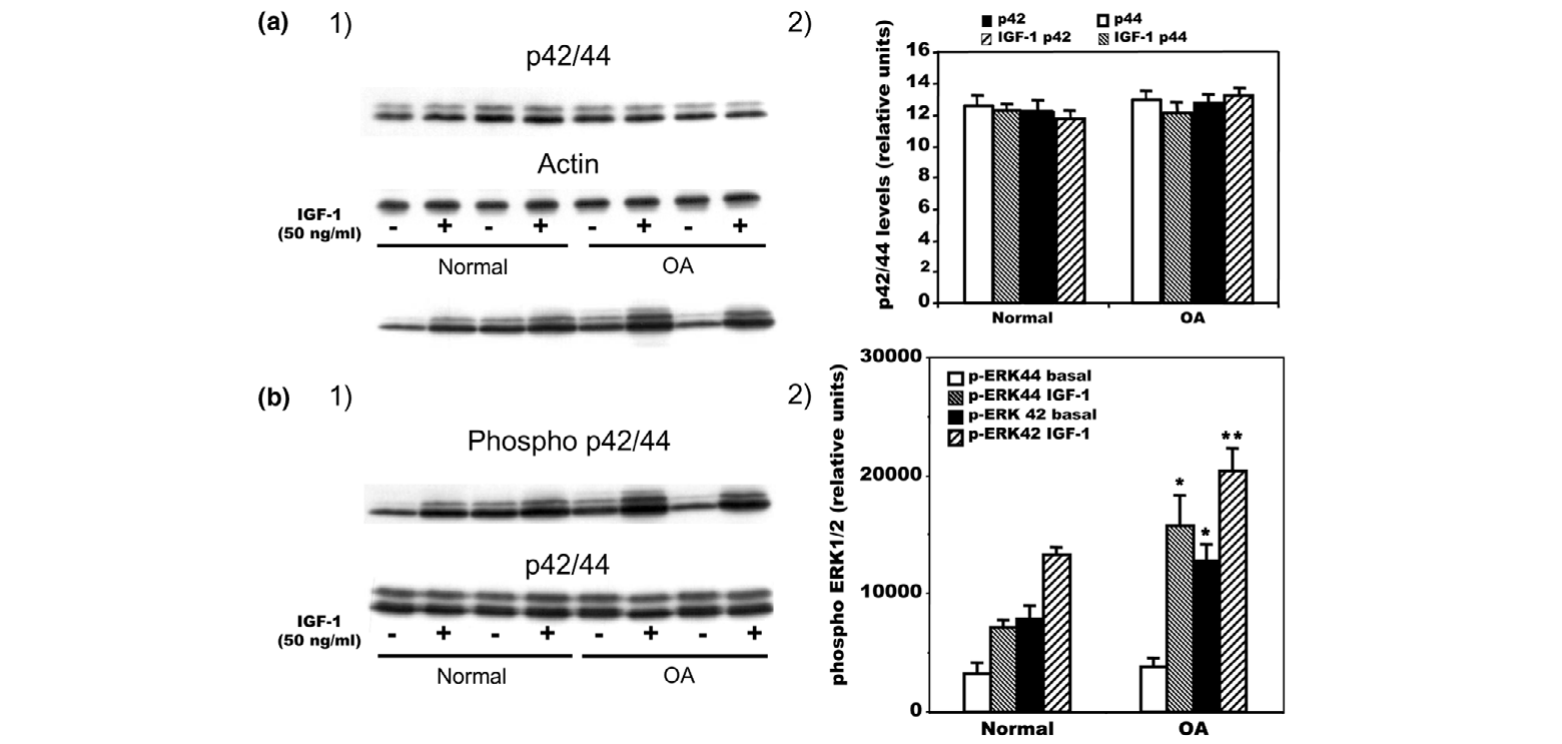
p42/44 levels and activation in normal and osteoarthitis (OA) osteoblasts. Cells were grown to confluence and incubated overnight in serum free medium. Cells were then exposed to 50 ng/ml insulin-like growth factor (IGF)-1 for 5 minutes. **(a) **After cell lysis in RIPA buffer p42/44 levels were visualized by immunoblotting with an anti-p42/44 antibody. **(b) **After cell lysis in RIPA buffer phosphorylated p42/44 levels were visualized by immunoblotting with an anti-phospho p42/44 antibody. Determination of actin was used as the control for loading. Representative data are shown in panel 1 (a,b). Results expressed as arbitrary scanning units are presented as the mean ± SEM (panel 2 (a,b)).

Since IGF-1 signaling involves the downstream activation of two different pathways, namely the MAPK and the PI3K pathways, we next evaluated if these pathways were altered similarly to IRS-1. We first looked at the protein levels of p42/44. Data showed no differences between normal (*n *= 3) and OA (*n *= 4) Obs (Figure 5a). In contrast, the phosphorylation of p42/44 was different between normal and OA Obs. After a 5 minute IGF-1 treatment, both p42 and p44 phosphorylation levels in OA Obs were increased by about 1.53 ± 0.15 (p < 0.025) and 2.18 ± 0.37 (p < 0.05) fold, respectively, compared to normal (Figure 5b). This increase in p42/44 phosphorylation is consistent with the observation that OA Obs grow faster than normal Obs (this study and [7]) and show reduced apoptosis (see above). Phosphorylation of p42/44 was sustained until 15 minutes, when a difference between normal and OA Obs could still be observed (data not shown). PI3K protein levels were unaltered between normal (*n *= 3) and OA Obs (*n *= 3) (data not shown). Similarly, PKB protein levels in normal (*n *= 3) and OA (*n *= 3) Obs were similar. Moerover, although the phosphorylation of PKB was slightly upregulated in OA (19.2 ± 1.0 relative units, *n *= 3) compared to normal (14.1 ± 1.5 relative units, *n *= 3) after a 5 minute IGF-1 stimulation, this difference did not reach statistical significance.

### Shc and Grb-2 protein levels and activation are similar in normal and osteoarthitis osteoblasts

**Figure 6:**
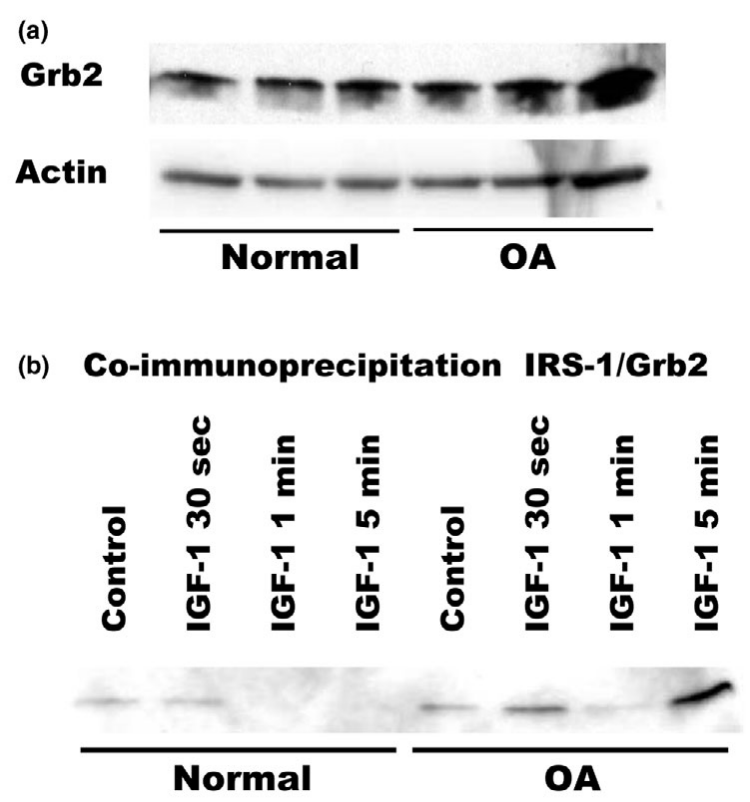
Grb2 levels and co-immunoprecipitation with insulin receptor substrate (IRS)-1 in normal and osteoarthitis (OA) osteoblasts. Cells were grown to confluence and incubated overnight in serum free medium. **(a) **After cell lysis in RIPA buffer Grb2 levels were visualized by immunoblotting with an anti-Grb2 antibody. The data show a representative immunoblot for three normal and three OA osteoblast preparations. **(b) **In another set of experiments, cells were exposed to 50 ng/ml insulin-like growth factor (IGF)-1 for various length of time or its vehicle, as indicated. After cell lysis in RIPA buffer and immunoprecipitation with an anti-IRS-1 antibody, co-immunoprecipitated Grb2 levels were visualized by immunoblotting with an anti-Grb2 antibody. A representative immunoblotting of four different experiments is presented. Determination of actin was used as the control for loading.

As Shc can also be recruited by IGF-1R and activate downstream SH2-domain containing proteins, we also evaluated the activation of this alternative pathway. No differences were found between total Shc protein levels in normal (*n *= 4) and OA Obs (*n *= 4). The Shc phosphorylation levels were also not significantly different between normal and OA Obs after a 5 minute IGF-1 stimulation (data not shown). This suggests that this pathway was unaltered in OA Obs. We also examined the levels of the small adaptor protein Grb2. Data illustrated in Figure 6a showed no difference between normal and OA Obs under basal conditions. Upon IGF-1 stimulation, Grb2 levels did not change either. However, as Grb2 can be associated with either Shc or phosphorylated IRS-1, we next evaluated the levels of Grb2 associated with the IRS-1 pathway using a co-immunoprecipitation strategy. As shown in Figure 6b, co-immunoprecipitation with IRS-1 followed by western blot detection of Grb2 showed weak Grb2 levels in both normal (*n *= 4) and OA (*n *= 4), although higher levels could be detected in OA Obs (4.76 ± 1.44-fold increased levels in OA Obs compared to normal, p < 0.05) under basal conditions. Grb2 levels associated with IRS-1 did not change significantly in normal Obs in response to 30 s of IGF-1 stimulation while the levels decreased with a longer incubation in the presence of IGF-1. In OA Obs, co-immunoprecipitated Grb2 levels increased slowly in response to IGF-1 stimulation; however, Grb2 levels reached about a 2.6 ± 0.8-fold increase compared to basal levels after a 5 minute IGF-1 stimulation (p < 0.03 by ANOVA) in OA Obs. A significant difference was also noted between Grb2 levels upon a 5 minute IGF-1 stimulation in OA Obs compared to normal (p < 0.01).

### Abnormal Syp modulation in OA Obs is implicated in underphosphorylation of IRS-1

**Figure 7:**
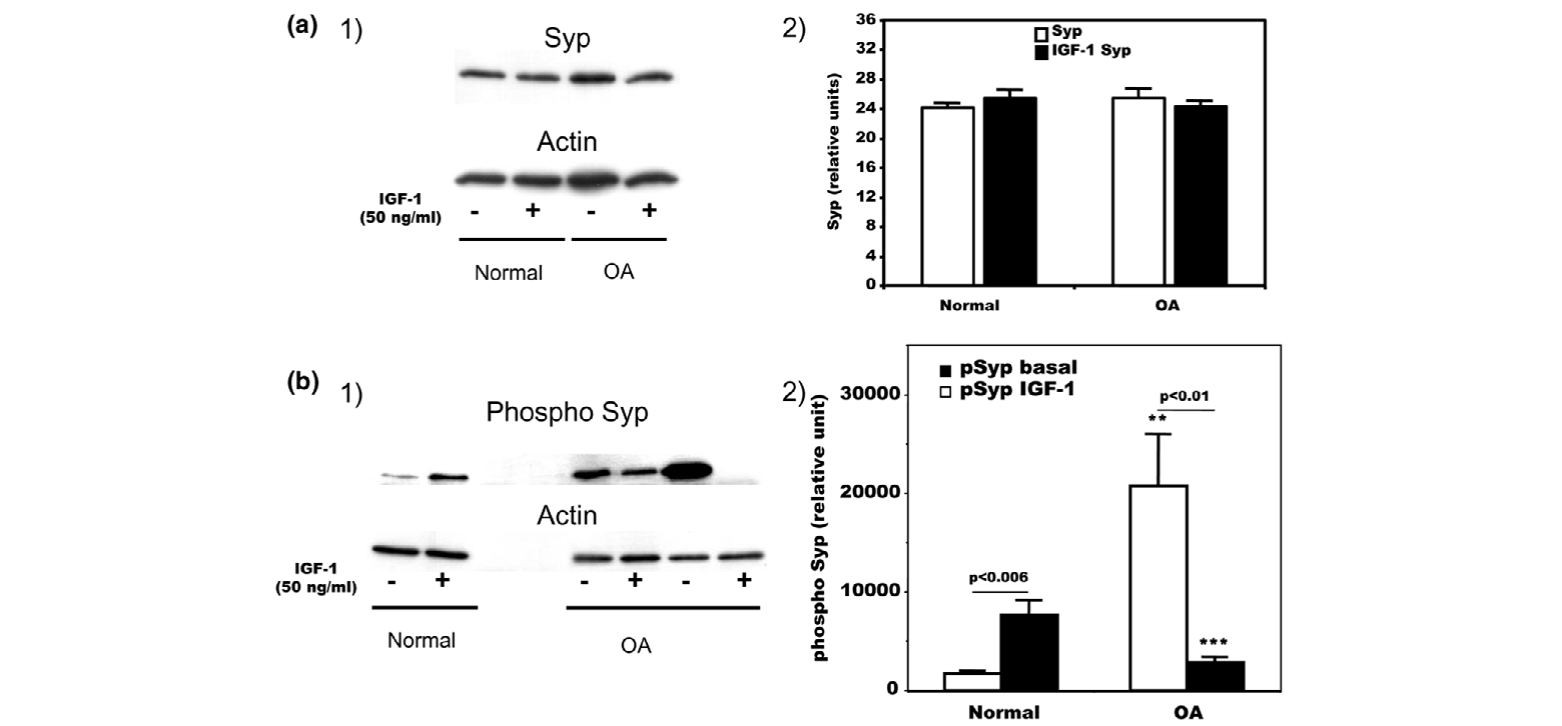
Syp activation in normal and osteoarthitis (OA) osteoblasts. Cells were grown to confluence and incubated overnight in serum free medium. Cells were then exposed to 50 ng/ml insulin-like growth factor (IGF)-1 for 5 minutes. **(a) **After cell lysis in RIPA buffer Syp levels were visualized by immunoblotting with an anti-Syp antibody. **(b) **After cell lysis in RIPA buffer and immunoprecipitation with an anti-phosphotyrosine antibody (PY-20), phosphorylated Syp levels were visualized by immunoblotting with an anti-Syp antibody. Determination of actin was used as the control for loading. Representative data are shown in panel 1 (a,b). Results expressed as arbitrary scanning units are presented as the mean ± SEM (panel 2 (a,b)).

**Figure 8:**
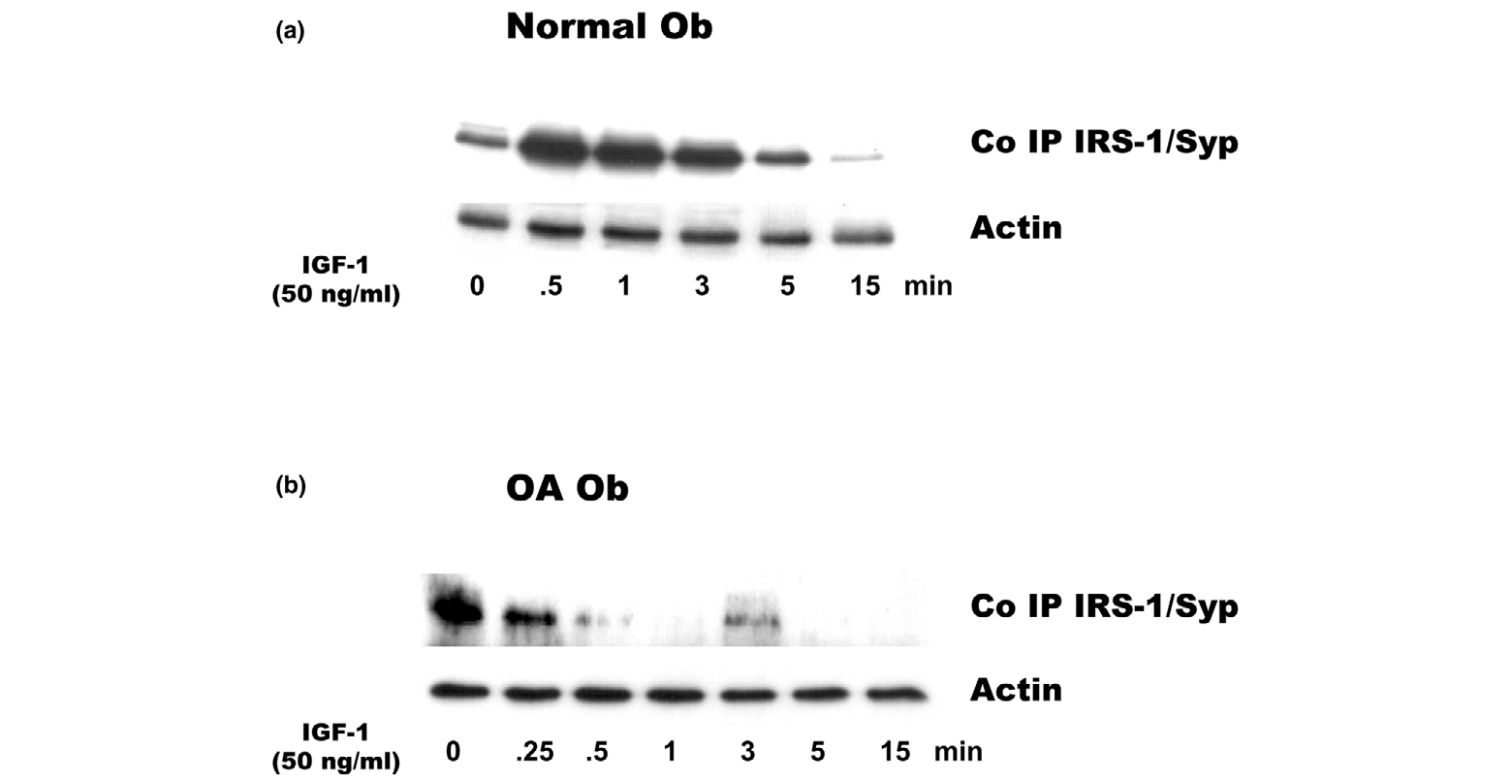
Co-immunoprecipitation (Co IP) of Syp with insulin receptor substrate (IRS)-1 in normal and osteoarthitis (OA) osteoblasts. Cells were grown to confluence and incubated overnight in serum free medium. Cells were then exposed to 50 ng/ml insulin-like growth factor (IGF)-1 for various lengths of time, as indicated. After cell lysis in RIPA buffer and IP with an anti-IRS-1 antibody, co-immunoprecipitated Syp levels were visualized by immunoblotting with an anti-Syp antibody. A representative immunoblotting of three different experiments is presented. Determination of actin was used as the control for loading.

Syp is a phosphatase known to bind IRS-1 and, once phosphorylated on its tyrosine residues, modulate its activity. We first compared Syp levels between normal and OA Obs. As observed with the other proteins, no differences were noted for Syp levels between normal (*n *= 3) and OA Obs (*n *= 4) as shown in Figure 7a. Surprisingly, time-dependent Syp phosphorylation in response to IGF-1 was clearly different between normal and OA Obs. As illustrated in Figure 7b, we observed the expected activation pattern with normal Obs (*n *= 3) in response to a 1 minute IGF-1 stimulation, increasing the phosphorylation levels of Syp by 2.7 ± 1.0-fold. On the other hand, in OA Obs (*n *= 5), basal Syp phosphorylation was already higher (12.23 ± 3.09 fold, p < 0.025) compared to normal. After a 1 minute IGF-1 stimulation, Syp phosphorylation in OA Obs decreased by 11.94 ± 5.93-fold, which reduced the phosphorylated Syp levels in OA Obs below those in normal Obs (p < 0.015). To demonstrate the implication of Syp phosphatase in the underphosphorylation of IRS-1 in the resting state, we next performed a series of co-immunoprecipitation assays to evaluate the interaction of Syp with IRS-1. As shown in Figure 8a, there was little Syp associated with IRS-1 in the basal state for normal Obs (*n *= 4). However, after a 30 s stimulation with IGF-1, there was a marked increased association of Syp with IRS-1 in normal Obs that returned to near basal state after 5 minutes. In OA Obs, the initial Syp levels associated with IRS-1 are higher than normal (approximately four-fold) and decreased rapidly to near undetectable levels upon a one minute IGF-1 stimulation (Figure 8b). These results suggest that the low IRS-1 phosphorylation levels observed in basal OA Obs could result from the increased association and activation of Syp phosphatase to IRS-1.

## Discussion

As an increasing amount of literature is demonstrating the involvement of bone tissue in the initiation/progression of OA, a better understanding of this tissue is clearly of utmost importance to better understand the etiology of this pathology. IGF-1 is one of the leading growth factors implicated in bone remodeling [31]. Interestingly, IGF-1 expression is increased in OA Obs [32] and these cells present an abnormal response to this growth factor [6,7]. Hence, we wanted to know if this is related to an alteration of its signaling pathway. We conducted a series of experiments to evaluate the IGF-1 signaling pathway in OA Obs and data revealed that the interaction of IRS-1 with Syp and Grb2 was modified in these cells in response to IGF-1 stimulation.

IGF-1R levels as well as phosphorylated IGF-1R levels following IGF-1 stimulation were similar in normal and OA Obs. However, IRS-1, the major IGF-1R docking protein, presented a reduced phosphorylation level in OA Obs, albeit the total protein level was similar between normal and OA Obs. In contrast, the IGF-1R docking protein Shc had similar protein and phosphorylation levels in normal and OA Obs. As IRS-1 was the only IGF-1R docking protein showing an abnormal modulation in OA Obs, we pursued our investigation with this factor and looked for molecules that could regulate IRS-1 phosphorylation.

First, 14.3.3 protein, which is known to bind IRS-1 and modulate its activation [19], was not significantly different between normal and OA Obs, and thus is unable to explain the underphosphorylation of IRS-1 in OA. Second, as the phosphorylation of both Shc and IRS-1 is linked to IGF-1R kinase activation and the phosphorylation of Shc was similar between normal and OA Obs, we can not conclude that an abnormal IGF-1R kinase activation explains the reduced phosphorylation of IRS-1. Third, we looked for phosphatases able to modulate IRS-1 activity. The best characterized phosphatase that binds to and modulates IRS-1 is Syp (or SHP-2) [33]. Syp protein levels were unaltered between normal and OA Obs. In contrast, its phosphorylation levels clearly demonstrated abnormal regulation in OA Obs. Indeed, OA Obs showed increased basal phosphorylation levels compared to normal, which was followed with a rapid decrease upon IGF-1 stimulation, in contrast to the situation for normal Obs. Moreover, the co-immunoprecipitation of Syp/IRS-1 also demonstrated an increased interaction in the basal state between IRS-1 and Syp in OA Obs, unlike in normal cells. Since there is an increase in IGF-1 production in OA Obs with a concomitant decrease of the major insulin-like growth factor binding proteins, namely BP-3, BP-4 and BP-5, OA Obs are likely to be more chronically stimulated by IGF-1 than normal Obs [32]. This suggests that the dowregulation of IRS-1 in OA Obs, the major IGF-1 signaling pathway, is a feedback response to increased exposure to elevated endogenous IGF-1 levels.

The observed increase in ERK1/2 phosphorylation, while there was no significant increase in PI3K activity measured by Akt/PKB phosphorylation, should promote an increase in cell proliferation. In this respect, it is noteworthy that primary OA Ob cell cultures grow faster than normal Obs [7], and we observed a reduction of the ratio of expression of Bax-α in OA Obs compared to normal. As Bax-α promotes apoptosis, a reduction in the Bax-α/Bcl2 ratio suggests an inhibition of apoptosis in OA Obs [30]. In the present study we also observed that OA Obs can grow faster than normal Obs and that they respond to IGF-1 stimulation with a greater cellular proliferation rate. This response to IGF-1 was strictly ERK1/2 dependent since PD98059 was able to fully inhibit the effect of IGF-1 on OA Obs. Such a situation could then lead to more cells being available and prolonged cell life that would possibly lead to the laying down of more extracellular matrix, as reported in OA subchondral bone tissue [4,5]. This also agrees with the recent demonstration that Obs from OA patients show enhanced proliferation and collagen type I expression *in vitro *compared to normal Obs [34]. However, addition of exogenous IGF-1 to OA Obs failed to increase collagen type I levels, which are already higher in these cells than normal. In contrast, as IGF-1 promoted alkaline phosphatase production by Obs, it is noteworthy that it stimulated this activity better in OA Obs than in normal Obs, and that this was also dependent on ERK1/2 activity. Similar observations were previously reported for both activities in Ob-like cells [35] and for cell proliferation alone in mesangial cells [36]. Overall, these data would then suggest that the activation of the ERK1/2 pathway in OA Obs in response to IGF-1 is important for cell proliferation, retards apoptosis and affects alkaline phosphatase. This could also promote the production of collagen type I overall as more cells would synthesize it, resulting in more collagen being laid down *in vivo*, although we could not show that more collagen was produced *per cell in vitro *in response to IGF-1. This is reminiscent of observations made in other tissues where IGF-1 alone could not promote collagen type I production but, in combination with other growth factors or high glucose levels, could do so [35-39].

One important question remains: if the main IGF-1R signaling pathway is dowregulated in OA Obs, how can we explain the increase in subchondral bone remodeling in OA bone tissue? Maybe the IGF-1 pathway is not implicated in this process. However, this seems unlikely since IGF-1 is a key regulator of bone remodeling and it increases uPA activity in OA Obs [6]. Once stimulated, the tyrosine kinase activity of IGF-1R leads to its autophosphorylation as well as the phosphorylation of a number of intracellular proteins, such as IRS-1, Shc and Gab1. This gives rise to the activation of Ras and PI3K, thus resulting in the activation of MAPK and PKB [40]. In our diseased cells, PI3K, MAPK and PKB protein levels were similar to normal, and we detected no significant increase in the activation of PKB while we observed a clear stimulation of the MAPK pathway. This implies the possibility of some complementary pathways for PKB activation that compensate for the lack of activation via IRS-1 in OA Obs. We recently demonstrated that transforming growth factor (TGF)-β production is increased in OA Obs [20] and, since this growth factor can activate PKB in arthritis, TGF-β stimulation could be one such compensatory mechanism activating PKB. Interestingly, IGF-1 dependent p42/44 stimulation was significantly increased in OA Obs. Since the activation of the Ras/MAPK pathway can modulate the production of uPA following growth factor stimulation, a situation we already observed in OA Obs [7], this suggests that the increased remodeling observed in OA subchondral bone could result from the upregulation of the p42/44 pathway following IGF-1 stimulation [41].

Increased activation of the p42/44 pathway concomitant with the dowregulation of IRS-1 in OA Obs may seem contradictory. One possible mechanism could be competition between IRS-1 and Shc for Grb2 [17]. In this model, IRS-1 and Shc compete for a limited cellular pool of Grb2, and the activation of the MAPK pathway would predominantly occur through the Shc-Grb2 signaling pathway. Grb2 is a small adaptor protein that can associate with IRS-1 and Shc via its SH2 domain and with the guanylnucleotide exchange factor for Ras, termed Son of Sevenless (SOS) via its SH3 domain. The association of the Grb2-SOS complex with tyrosine phosphorylated receptors and/or Shc have been directly implicated in the activation of the Ras signaling pathway. Since in OA Obs Grb2 levels were similar to normal, the downregulation of IRS-1 phosphorylation following IGF-1 stimulation in these diseased cells may result in an increased availability of Grb2 to the Shc pathway, leading to increased activity of the p42/44 pathway. However, as shown here, the interaction of Grb2 with IRS-1 was also increased in OA Obs, implying that the Grb2-Shc interaction should be reduced in these cells. On the other hand, the p42/44 kinase activity could also be activated directly by TGF-β via the Smad3 signaling pathway, as previously proposed by Sowa and colleagues [42], a situation that overules Grb2-Shc signaling. Indeed, as OA Obs have elevated TGF-β levels [20], this could directly activate the p42/44 pathway without the involvement of Grb2. Thus, the elevated endogenous TGF-β levels in OA Obs could then explain both the results for the PKB and p42/44 pathways observed here.

Taken together, these results could be interpreted as a general downregulation of the IGF-1R/IRS-1 pathways in OA Obs. However, dowstream signals were not actually reduced. The observed increase in Syp/IRS-1 interaction and increased Syp phosphorylation could actually promote IGF-1 signaling. A functional and highly phosphorylated SHP-2/Syp is necessary for sustained activation of ERK1/2 response to hepatocyte growth factor (HGF) stimulation in Madin-Darby canine kidney (MDCK) cells [43] and in rat fibroblasts in response to insulin, IGF-1 or epidermal growth factor [44]. Moreover, inactivating Syp antibodies [44] or expression of a mutant phosphatase [45] significantly reduces insulin, IGF-1 and epidermal growth factor signaling. Accordingly, as OA Obs showed high phosphorylated Syp levels and strong interaction with IRS-1, both under basal conditions and after IGF-1 stimulation, this could promote p42/44 activity in OA Obs, as observed in those studies. Moreover, a recent study indicated that functionally deficient SHP-1 mice are markedly glucose tolerant and insulin sensitive as a result of enhanced insulin receptor signaling to IRS-1 [46], which suggests that elevated activity of SHP-1/Syp could reduce IGF-1 signaling to IRS-1, as observed in the present study. This would also suggest that, although IGF-1R-dependent IRS-1 phosphorylation is reduced in OA Obs, Syp phosphorylation and activity could compensate for this reduction. As Syp is central to other growth factors, such as HGF and epidermal growth factor [43,44], and since we recently showed a key role for HGF in OA Obs [47] and possibly for the cross-talk between OA Obs and cartilage tissue [48], the present results suggest that the HGF-dependent pathway could also be altered in OA Obs, a situation not investigated at present.

## Conclusion

This is the first study demonstrating abnormal IGF-1 cell signaling in human OA subchondral Obs that could explain the abnormal response of these cells to this growth factor. We demonstrated an altered IGF-1 pathway involving IRS-1 and Syp. Moreover, we also demonstrated increased MAPK activity in IGF-1 stimulated OA Obs, which could be implicated in the abnormal subchondral bone remodeling observed in OA. Since both Syp and Grb2 play key roles in the signaling pathways of other growth factors in Obs besides IGF-1, this may also suggest an abnormal response to these growth factors in OA Obs.

## Abbreviations

BrdU = bromodeoxyuridine; BSA = bovine serum albumin; FBS = fetal bovine serum; GAPDH = glyceraldehyde-3-phosphate dehydrogenase; HGF = hepatocyte growth factor; IGF = insulin-like growth factor; IGF-1R = IGF-1 receptor; IRS = insulin receptor substrate; OA = osteoarthitis; Ob = osteoblast; PI3K = phosphatidylinositol 3-kinase; PKB = protein kinase B; SD = standard deviation; SEM = standard error of the mean; TGF = transforming growth factor; UNG = uracil-N-glycosylase; uPA = urokinase plasminogen activator.

## Competing interests

The authors declare that they have no competing interests.

## Authors' contributions

FM performed most of the experiments and wrote the first draft of the manuscript. IA performed the experiments shown in Figure 4 and contributed to writing the manuscript. JM-P and J-PP contributed to writing the manuscript and discussion of the results. JCF provided the OA knee samples and contributed to discussion of the results. DL proposed original concepts, planned and performed some of the experiments, performed the statistical analyses, participated in the discussion and wrote the final version of the manuscript.

## Supplementary Material

Additional file 1 A figure showing p42/44 levels and activation in normal and osteoarthitis (OA) osteoblasts. Cells were grown to confluence and incubated overnight in serum free medium. Cells were then exposed to 50 ng/ml insulin-like growth factor (IGF)-1 for 15 minutes. Phospho p42/44 levels were detected by westen blot analysis. The figure shows a representative experiment with one normal and one OA osteoblast preparation. Similar assays were repeated with three different samples of normal and OA osteoblasts with similar results. Click here for file

Additional file 2 A figure showing phospho-protein kinase B (pPKB) and phosphatidylinositol 3-kinase (PI3K) levels and activation in normal and osteoarthitis (OA) osteoblasts. Cells were grown to confluence and incubated overnight in serum free medium. Cells were then exposed to 50 ng/ml insulin-like growth factor (IGF)-1 for 5 minutes. The top panel shows a representative western blot of pPKB and total PKB levels in one normal and one OA osteoblast preparation. Similar assays were repeated with four different samples of normal and OA osteoblasts with similar results. The bottom panel shows a representative western blot of PI3K levels for two normal and two OA osteoblast preparations. Actin levels were determined to ensure similar loading between samples. Similar assays were repeated with four different samples of normal and OA osteoblasts with similar results. Click here for file
