# Supplementary figures and images for: Abnormal insulin-like growth factor 1 signaling in human osteoarthritic subchondral bone osteoblasts
Source: Arthritis Res Ther. 2006 Nov 27;8(6):R177. doi: 10.1186/ar2087 (PMC1794522; doi:10.1186/ar2087)

Supplemental Figure 1: Massicotte et al

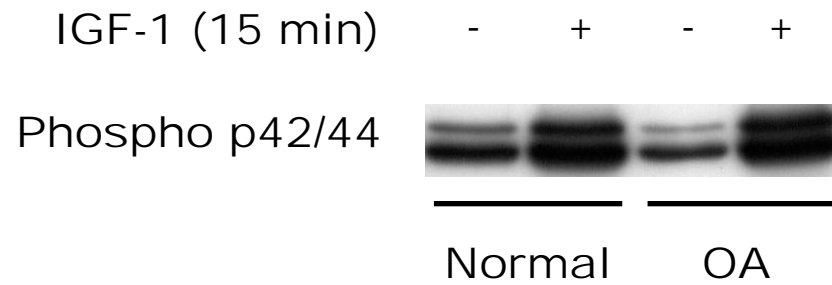

Supplement: Additional file 1 — A figure showing p42/44 levels and activation in normal and osteoarthitis (OA) osteoblasts. Cells were grown to confluence and incubated overnight in serum free medium. Cells were then exposed to 50 ng/ml insulin-like growth factor (IGF)-1 for 15 minutes. Phospho p42/44 levels were detected by westen blot analysis. The figure shows a representative experiment with one normal and one OA osteoblast preparation. Similar assays were repeated with three different samples of normal and OA osteoblasts with similar results. [file ar2087-S1.pdf]

Supplemental Figure 2: Massicotte et al

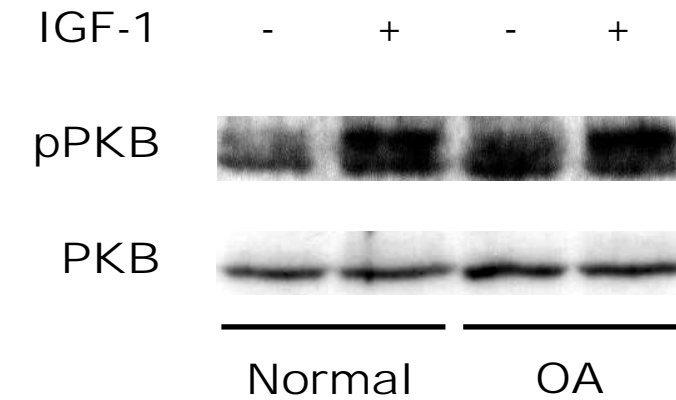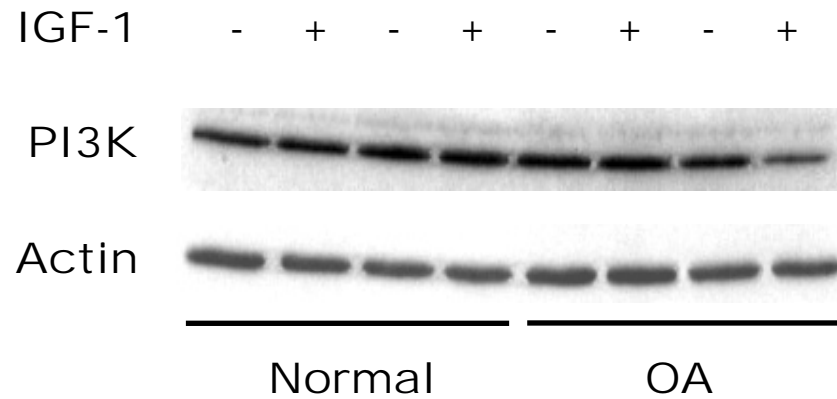

Supplement: Additional file 2 — A figure showing phospho-protein kinase B (pPKB) and phosphatidylinositol 3-kinase (PI3K) levels and activation in normal and osteoarthitis (OA) osteoblasts. Cells were grown to confluence and incubated overnight in serum free medium. Cells were then exposed to 50 ng/ml insulin-like growth factor (IGF)-1 for 5 minutes. The top panel shows a representative western blot of pPKB and total PKB levels in one normal and one OA osteoblast preparation. Similar assays were repeated with four different samples of normal and OA osteoblasts with similar results. The bottom panel shows a representative western blot of PI3K levels for two normal and two OA osteoblast preparations. Actin levels were determined to ensure similar loading between samples. Similar assays were repeated with four different samples of normal and OA osteoblasts with similar results. [file ar2087-S2.pdf]
